# Modulation of STAT3 Folding and Function by TRiC/CCT Chaperonin

**DOI:** 10.1371/journal.pbio.1001844

**Published:** 2014-04-22

**Authors:** Moses Kasembeli, Wilson Chun Yu Lau, Soung-Hun Roh, T. Kris Eckols, Judith Frydman, Wah Chiu, David J. Tweardy

**Affiliations:** 1 Section of Infectious Diseases, Department of Medicine, Baylor College of Medicine, Houston, Texas, United States of America; 2 Verna and Marrs McLean Department of Biochemistry and Molecular Biology, Baylor College of Medicine, Houston, Texas, United States of America; 3 Department of Biology and the BioX Program, Stanford University, Stanford, California, United States of America; 4 Department of Cellular and Molecular Biology, Baylor College of Medicine, Houston, Texas, United States of America; Whitehead Institute, United States of America

## Abstract

Signal transducer and activator of transcription 3 (Stat3) transduces signals of many peptide hormones from the cell surface to the nucleus and functions as an oncoprotein in many types of cancers, yet little is known about how it achieves its native folded state within the cell. Here we show that Stat3 is a novel substrate of the ring-shaped hetero-oligomeric eukaryotic chaperonin, TRiC/CCT, which contributes to its biosynthesis and activity *in vitro* and *in vivo*. TRiC binding to Stat3 was mediated, at least in part, by TRiC subunit CCT3. Stat3 binding to TRiC mapped predominantly to the β-strand rich, DNA-binding domain of Stat3. Notably, enhancing Stat3 binding to TRiC by engineering an additional TRiC-binding domain from the von Hippel-Lindau protein (vTBD), at the N-terminus of Stat3, further increased its affinity for TRiC as well as its function, as determined by Stat3's ability to bind to its phosphotyrosyl-peptide ligand, an interaction critical for Stat3 activation. Thus, Stat3 levels and function are regulated by TRiC and can be modulated by manipulating its interaction with TRiC.

## Abstract 2

Levels, folding, and function of the infamous cancer and inflammatory disease-related signaling molecule Stat3 are regulated by interaction with the chaperonin TRiC; manipulation of this interaction is a therapeutic avenue for exploration.

## Author Summary

Stat3 is a multidomain transcription factor that contributes to many cellular functions by transmitting signals for over 40 peptide hormones from the cell surface to the nucleus. Understanding how multidomain proteins achieve their fully folded and functional state is of substantial biological interest. As Stat3 signaling is up-regulated in many pathological conditions, including cancer and inflammatory diseases, insight into what controls its folding may be useful for the identification of vulnerabilities that can be therapeutically exploited. We demonstrate that the major protein-folding machine or chaperonin within eukaryotic cells, TRiC/CCT, is required for Stat3 to fold during its synthesis and for Stat3 to be fully functional within the cell. We also find that TRiC can refold chemically denatured Stat3 and provide evidence that the CCT3 subunit of TRiC binds to the DNA-binding domain of Stat3. We also show that Stat3 activity is decreased by down-modulating levels of TRiC and can be increased by increasing Stat3's interaction with TRiC. TRiC therefore regulates both Stat3 protein levels and its function, making Stat3 modulation by manipulation of its interaction with TRiC a potential approach for the treatment of cancer and inflammatory diseases.

## Introduction

Signal transducer and activator of transcription 3 (Stat3) is a member of a family of seven closely related proteins responsible for the transmission of peptide hormone signals from the extracellular surface of cells to the nucleus [1],[2]. Mice deficient in Stat3 died during early embryogenesis, indicating its critical role in development [3]. Conditional gene targeting of Stat3 in several types of tissues revealed important roles for Stat3 in key biological functions including cell survival and growth, immunity, and inflammation [3],[4]. These findings in mice have been recapitulated, in part, in patients with autosomal-dominant hyper-IgE syndrome (AD-HIES) or Job's syndrome, a rare immunodeficiency syndrome, which results from loss-of-function mutations in the Stat3 gene [5]. At the opposite end of the spectrum, gain-of-function mutations or increased wild-type Stat3 activity has been implicated in the pathogenesis of up to 50% of hematological and solid tumors [6]–[8]. Despite its importance to normal physiology and pathophysiology, however, little is known about how Stat3 achieves its native state within the cell, information that potentially could be exploited to develop novel therapies for AD-HIES and/or cancer.

For the majority of eukaryotic proteins, folding to the native and functional conformation requires the assistance of elaborate cellular machinery composed of proteins known as molecular chaperones [9],[10]. The chaperonins comprise a class of oligomeric, double-ring, high molecular weight, ATP-dependent chaperones with the unique ability to fold certain proteins that cannot be folded by simpler chaperone systems [11],[12]. The primary cytosolic eukaryotic chaperonin is the tailless complex protein-1 (TCP-1) ring complex (commonly abbreviated TRiC, and also known as complex containing TCP-1 or CCT). TRiC is a large complex (1 MDa) composed of eight homologous but distinct subunits (CCT 1–8), arranged in two stacked octameric rings to form two interior chambers in which substrate proteins can be encapsulated and folded. TRiC is essential for the *de novo* folding of approximately 10% of newly synthesized proteins in the eukaryotic cell and for refolding proteins that become denatured following stress [13]. These substrate proteins extend up to 120 kDa in size, and most appear to contain regions with β-strands [14]. Well-characterized clients of TRiC include the cytosolic proteins actin, tubulin, and the tumor-suppressor von Hippel-Lindau protein (pVHL). In contrast, details regarding the contribution of TRiC to the folding and function of transcription factors are limited.

Here we demonstrate that TRiC binds the oncogenic transcription factor, Stat3, and contributes to its biosynthesis, refolding, and activity *in vitro* and within cells. TRiC binding to Stat3 was mediated, at least in part, by TRiC subunit CCT3. Stat3 binding to TRiC mapped primarily to its β-strand-rich, DNA-binding domain (DBD). Addition of a second TRiC-binding domain (TBD) to the N-terminus of Stat3 (the TBD of pVHL, vTBD) further increased the affinity of Stat3 for TRiC and its function. Thus, the structure and function of Stat3 is regulated by the major eukaryotic chaperonin TRiC/CCT and can be modulated through manipulation of TRiC levels or substrate affinity.

## Results

### Stat3 Is a Member of the TRiC Interactome and Requires TRiC for Its Biogenesis and Optimal Activity

**Figure 1:**
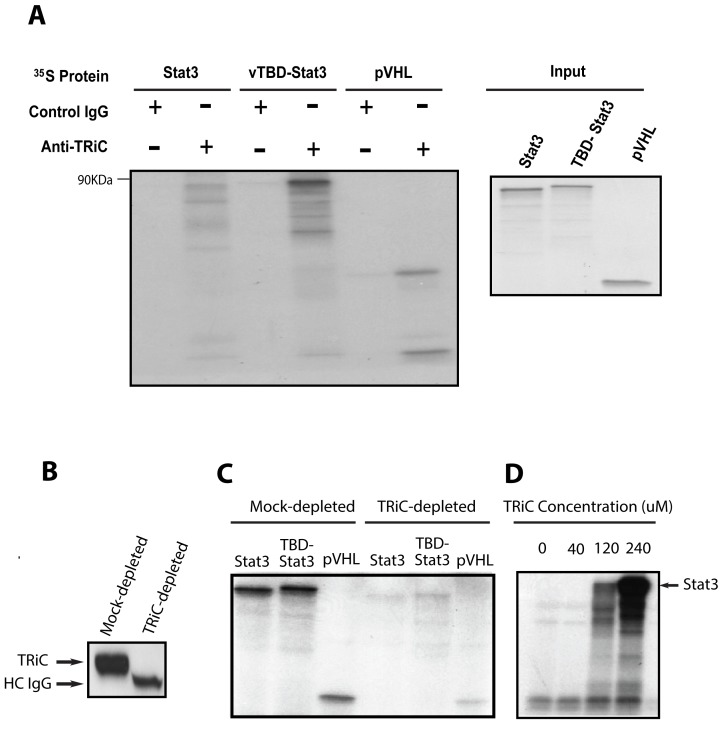
TRiC binds Stat3 co-translationally and is required for its synthesis in RRLs. In panel (A), TRiC was immunoprecipitated from RRLs with a combination of antibodies to CCT2 and CCT5 (Anti-TRiC) or with a nonspecific control antibody (Control) following translation of the indicated proteins in the presence of ^35^S-methionine. Immunoprecipitates were separated by SDS-PAGE and autoradiographed (left panel); the position of the 90-kDa MW marker is indicated. Half of each IP reaction prior to precipitation was run separately on SDS-PAGE and autoradiographed (right panel). In panel (B), RRLs were immunoblotted with CCT1 antibody following TRiC depletion using Protein-A agarose plus CCT1 antibody (TRiC-depleted) or control antibody (Mock-depleted). In panel (C), the indicated protein was translated in mock-depleted or TRiC-depleted RRLs in the presence of ^35^S-methionine followed by SDS-PAGE and autoradiography. In panel (D), Stat3 was translated in TRiC-depleted RRLs following the addition of purified bovine TRiC in increasing amounts in the presence of ^35^S-methionine followed by SDS-PAGE and autoradiography. The results shown are representative of three experiments.

To determine if Stat3 interacts with TRiC, we performed TRiC immunoprecipitation in a cell-free mammalian translation system used previously [13] to identify new members of the TRiC interactome (rabbit reticulocyte lysates, RRLs). Similar to pVHL, radiolabeled Stat3, both full-length and nascent polypeptide chains, immunoprecipitated with TRiC (Figure 1A), indicating that Stat3 is a member of the TRiC interactome and suggesting that its interaction with TRiC occurs co-translationally, as described previously for other TRiC client proteins [15].

To assess if the TRiC interaction with nascent Stat3 polypeptides is coupled to their biogenesis, RRLs were immunodepleted of TRiC and tested for the ability to generate Stat3. TRiC depletion of RRLs (Figure 1B) markedly reduced its ability to generate Stat3 (Figure 1C). Importantly, add-back of purified bovine TRiC to TRiC-depleted RRL reconstituted Stat3 protein synthesis in a dose-dependent manner (Figure 1D). These findings indicate that TRiC is required for Stat3 protein biogenesis within RRLs and suggests that the co-translational interaction TRiC with Stat3 is coupled to Stat3 biogenesis.

**Figure 2:**
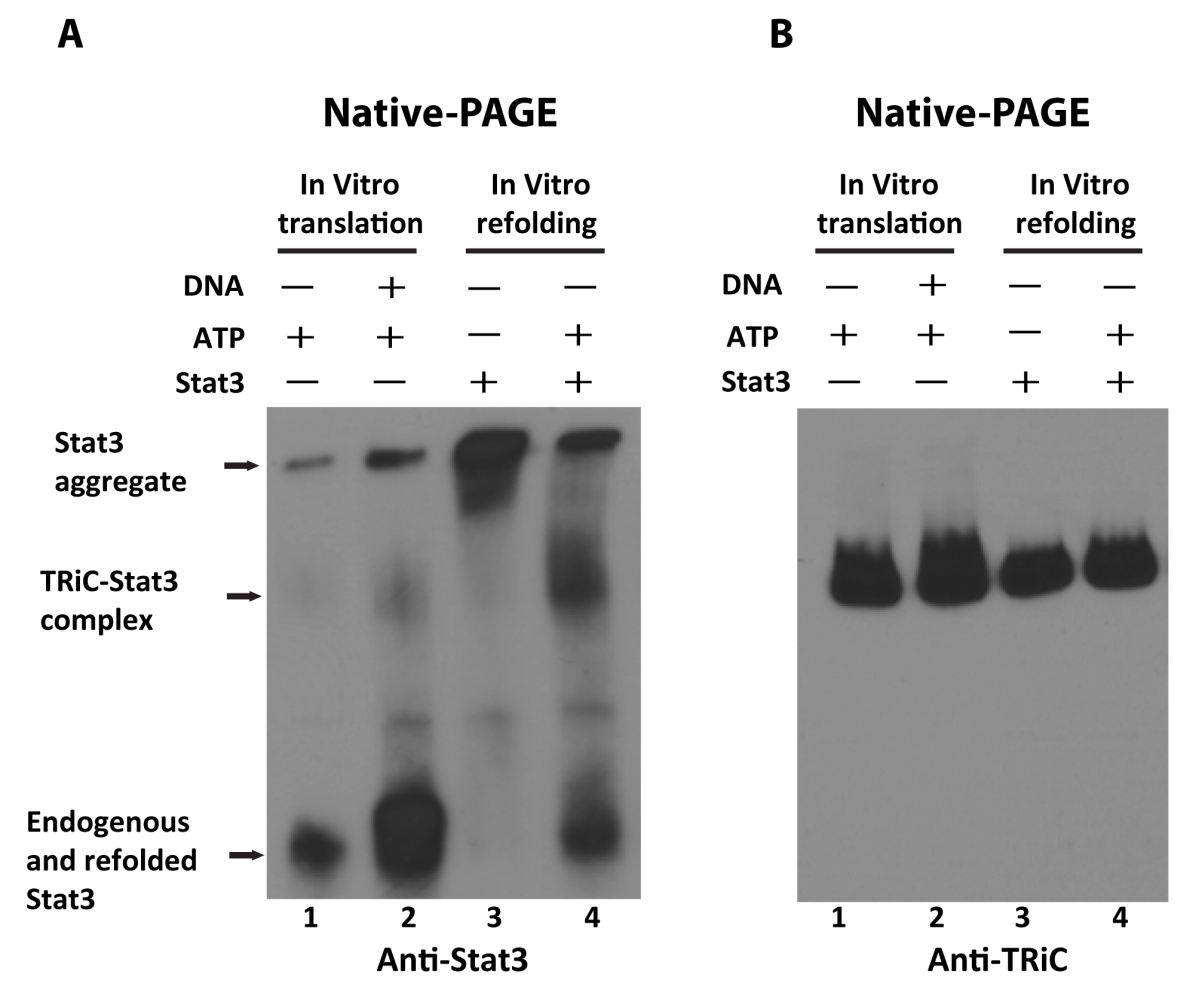
Formation of TRiC-Stat3 complexes and refolding of guanidine hydrochloride-denatured Stat3 within RRLs containing ATP. In panel (A), Stat3-containing complexes were detected within RRLs following native-PAGE analysis of *in vitro* translation and refolding reaction products by Western blot using an anti-Stat3 antibody. Lane 1 shows RRL reaction in the absence of addition of exogenous plasmid. Lane 2 shows RRL reaction in the presence of addition of exogenous plasmid encoding Stat3. Stat3 previously unfolded using 6 M guanidine hydrochloride was added to RRLs without ATP (lane 3) or RRLs containing ATP (lane 4). Arrows indicate the position of Stat3 aggregate, TRiC-Stat3 complex, and native endogenous and refolded Stat3. In panel (B), the blot was stripped and re-probed with anti-CCT1 antibody.

In addition to folding newly synthesized proteins, TRiC also functions to refold native proteins within the cell that become denatured under stress. To determine if TRiC refolds unfolded Stat3 *in vitro*, Stat3 protein chemically denatured in guanidine hydrochloride was added to RRLs before and after ATP depletion and the levels of refolded Stat3 assessed by native PAGE (Figure 2). RRL extracts before and after *in vitro* Stat3 translation were run on the same gel to indicate the location of native or refolded Stat3 and aggregated Stat3. RRLs contained low levels of native endogenous Stat3, which, as expected, increased markedly following *in vitro* translation (Figure 2A, lanes 1 and 2). Also, a small amount of endogenous Stat3 is unable to enter the gel, indicative of aggregate formation; *in vitro* translation increased the amount of aggregate detected, in addition to the amount of native folded Stat3. Addition of denatured Stat3 into RRLs depleted of ATP resulted in a marked increase in the amount of Stat3 aggregate detected, as well as a decrease in the levels of endogenous native Stat3 detected (Figure 2A, lane 3); the latter is most likely due to co-aggregation of native endogenous Stat3 with exogenous denatured Stat3. In contrast, addition of denatured Stat3 to RRLs not depleted of ATP resulted in the reappearance of folded Stat3 and a marked decrease in Stat3 aggregate detected (Figure 2A, lane 4). Thus, TRiC-containing RRLs are capable of folding denatured Stat3 in the presence of ATP. In addition to bands representing native Stat3 and aggregated Stat3, a third Stat3-containing band was detected particularly within the RRL samples containing the greatest levels of folded Stat3 (Figure 2A, lanes 2 and 4). Immunoblotting indicated that this Stat3-containing band co-migrated with TRiC (Figure 2B), indicating that it most likely represents a complex of TRiC binding to folding intermediates of Stat3. Of note, TRiC does not bind to aggregates of Stat3 (Figure 2A, lane 3; Figure 2B, lane 3), in contrast to aggregated proteins containing expanded polyglutamine repeats (Tam et al., 2006) [16]. These results strongly suggest that, in addition to *de novo* Stat3 protein folding, TRiC within RRL participates in the refolding of denatured Stat3 protein *in vitro*.

**Figure 3:**
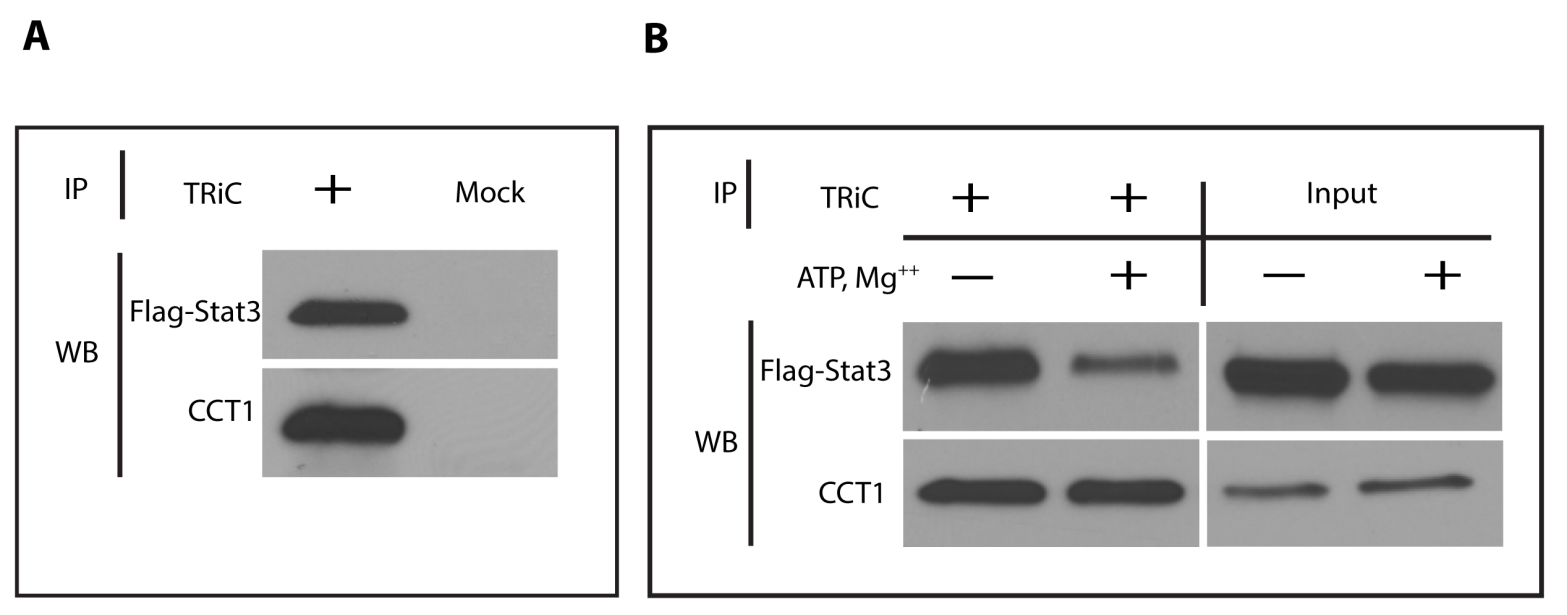
Stat3 interacts with TRiC within MEF cells. In panel (A), lysates of MEF-Stat3^Δ/Δ^ cells transiently expressing Flag-Stat3α were immunoprecipitated (IP) with a mixture of rabbit antibodies to CCT2 and CCT5 (+) or control rabbit antibody to human IgG (Mock) and Western blotted (WB) with antibodies to Flag tag or CCT1. In panel (B), TRiC lysates of MEF-Stat3^Δ/Δ^ cells transfected with Flag-tagged Stat3α were prepared in the presence or absence of ATP and MgCl_2_, and analyzed by immunoprecipitation and Western blotting. The input is shown to the right.

Having established that TRiC interacts with newly translated and denatured Stat3 in RRLs, promoting its synthesis and folding *in vitro*, we asked if this interaction could be detected *in vivo* within mammalian cells. We transfected murine embryonic fibroblast cells in which Stat3 was deleted using Cre-Lox technology (MEF/Stat3^Δ/Δ^ cells) [17] with Flag-tagged Stat3. TRiC was immunoprecipitated from lysates of these cells in the absence or presence of exogenous ATP (2 mM). Stat3 co-immunoprecipitated with TRiC in the absence of ATP (Figure 3A and B). In addition, the amount of Stat3 that co-immunoprecipitated with TRiC was reduced substantially in the presence of ATP (Figure 3B), indicating that, similar to other TRiC clients, the interaction of Stat3 with TRiC is ATP dependent.

**Figure 4:**
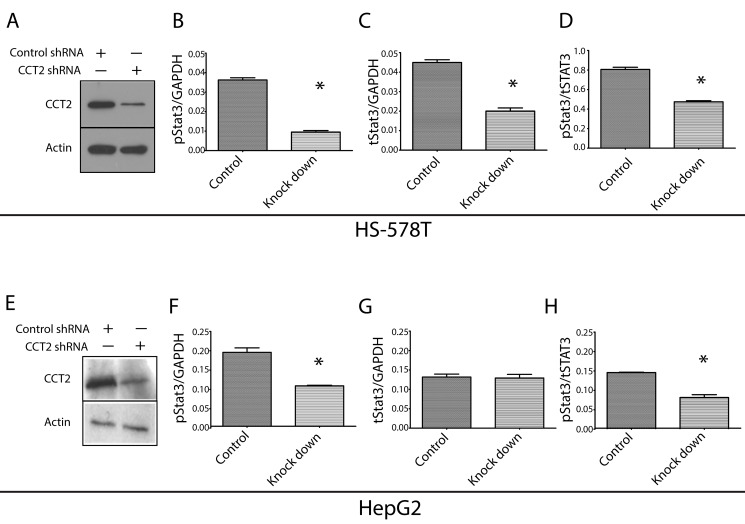
TRiC knockdown reduces levels of activated Stat3 protein within cancer cells. HS-578T cells (A) or HepG2 cells (E) stably transfected with lentivirus containing CCT2 shRNA (knockdown) or control shRNA (control) were immunoblotted with antibody to CCT2 or β-actin. Levels of activated Stat3 (Y705-phosphorylated Stat3, pStat3), total (t)Stat3, and GAPDH within cells lysates were measured using Luminex beads. Levels of pStat3 in HS-578T and HepG2 CCT2 or control knockdown cells are presented normalized to GAPDH (B and F) or tStat3 (D and H); levels of tStat3 in HS-578T and HepG2 CCT2- or control-knockdown cells are presented normalized to GAPDH (C and G). Results in CCT2 knockdown cells indicated with an asterisk (*) differ from control knockdown cells (*p*<0.02). The results shown are representative of two experiments.

To determine the contribution of TRiC to the levels of total and activated Stat3 (Stat3 phosphorylated on Y705, pStat3) within cells, we used shRNA targeting CCT2 to reduce the levels of TRiC within two cells lines (HS-578T and HepG2), both previously demonstrated to have constitutively activated Stat3 [18],[19]. Immunoblotting demonstrated 90% and 70% reduction of TRiC CCT2 in HS-578T and HepG2 cells, respectively, following stable transfection with CCT2 shRNA vector compared to control cells (Figure 4A and 4E). In addition to reduction in levels of CCT2, other CCT subunits not targeted by shRNA (CCT1 and 5) were similarly decreased in CCT2 knockdown cells (Figure S1), confirming previous observations that excess CCT subunits not within TRiC complexes are degraded [20].

Knockdown of TRiC in HS-578T cells decreased constitutively activated Stat3 (pStat3) levels by 74% (*p* = 0.0026; Figure 4B). The decrease in pStat3 levels resulted from both a decrease in the pool of total Stat3, which was decreased by 41% (*p* = 0.0056; Figure 4C), and a decrease in the proportion of total Stat3 that was phosphorylated, which was decreased by 56% (*p* = 0.0072; Figure 4D). Similarly, knockdown of TRiC in HepG2 cells decreased pStat3 levels by 46% (*p* = 0.0176; Figure 4F). In contrast to HS-578T, however, there was no decrease in total Stat3 (Figure 4G); rather, the decrease in pStat3 levels was due entirely to a 44% decrease in the proportion of total Stat3 that was phosphorylated (*p* = 0.0124; Figure 4H). Thus, levels of constitutively activated Stat3 (pStat3) and total Stat3 are decreased in cells in which TRiC levels are reduced; pStat3 levels are more sensitive to reduced TRiC than total Stat3 levels, which appear to require 90% or greater reduction in TRiC to be affected.

**Figure 5:**
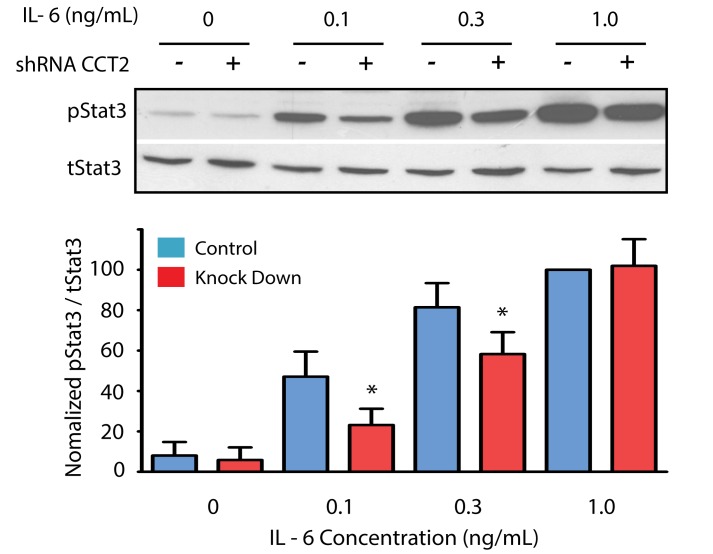
TRiC knockdown reduces sensitivity of cancer cells to IL-6–mediated Stat3 activation. CCT2-knockdown (+) or control-knockdown (−) HepG2 cells were immunoblotted for pStat3 and total Stat3 (tStat3) 30 min after incubation in media alone or in media containing IL-6 at the indicated concentrations. Representative immunoblots are shown in the top panel. Densitometry readings of the immunoblot bands were normalized to the IL-6 concentration yielding maximum pStat3 levels (1 ng/ml) in two experiments. The ratios of densitometry values (pStat3/tStat3) for these experiments is shown in the bottom panel; results in CCT2 knockdown cells that are indicated with an asterisk (*) differ from control knockdown cells (*p*<0.05).

To determine if levels of cytokine-activated Stat3 are affected by TRiC reduction in addition to constitutively activated Stat3, we examined the levels of pStat3 in IL-6 stimulated control and TRiC knockdown HepG2 cells. Use of the HepG2 control and TRiC knockdown cells, as opposed to their HS-578T counterparts, allowed us to examine the levels of cytokine-activated Stat3 independent of levels of total Stat3. HepG2 cells were treated for 30 min with increasing concentrations of IL-6 in the most sensitive portion of the dose–response curve (0, 0.1, 0.3, and 1 ng/ml) (Figure 5). Levels of pStat3 in knockdown cells normalized to total Stat3 were reduced at 0.1 ng/ml and 0.3 ng/ml IL-6 concentrations (*p*<0.05 for both). These results indicated that TRiC contributes to maximal levels of ligand-activated Stat3 within cells at sub-maximal ligand concentrations. Thus, levels of both constitutively activated and ligand-activated Stat3 are sensitive to decreased TRiC levels within cells.

### The TBD within Stat3 Maps Predominantly to Its DBD

**Figure 6:**
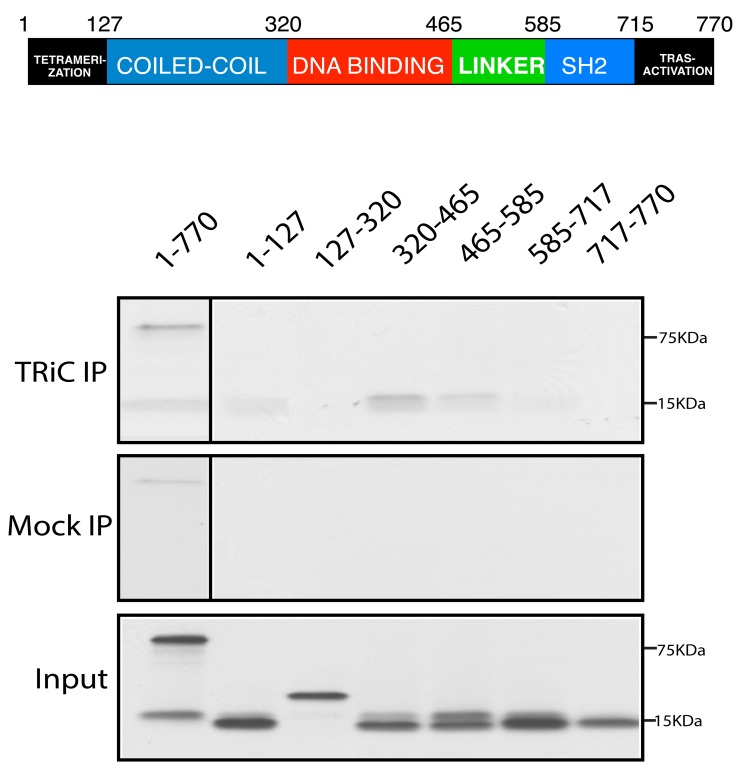
The TBD within Stat3 maps predominantly to its DBD. A schematic depiction of the six Stat3 domains is shown in the top portion of the Figure. In the bottom portion, TRiC was immunoprecipitated using a combination of antibodies to CCT2 and CCT5 (TRiC IP) or control antibody (Mock IP) from RRL transcription/translation reactions containing expression constructs with inserts encoding each of six domains: the N-terminal oligomerization/tetramerization domain, the DBD, the linker domain, the SH2 domain, or the transactivation domain. TRiC and mock immunoprecipitates and an equal volume of RRL from each reaction mixture (Input) were separated by SDS-PAGE and autoradiographed. The results shown are representative of more than three experiments.

Stat3 consists of six distinct functional domains (oligomerization or tetramerization, coiled-coil, DNA-binding, linker, Src-homology 2, and transactivation; Figure 6) [2],[21]. X-ray crystallography of the core Stat3 protein established the structural features of the four central domains [22] and demonstrated that two of the four domains (DNA binding and Src-homology 2) contain β strands, the structural motif within TRiC client proteins shown previously to be preferentially bound by TRiC [23],[24]. To identify Stat3 domains that participate in TRiC binding, we performed TRiC immunoprecipitation from RRLs that expressed individual Stat3 domains. Of the individual domains, the DBD had the strongest association with TRiC (Figure 6) followed by the linker domain, the N-terminal domain, and the SH2 domain. Fifty-seven percent of the secondary structure of the Stat3 DBD consists of β strands, which form a β barrel similar to the DBD of other members of the NF-κB/NF-AT superfamily of which Stat3 is a member. Thus, the Stat3 TBD maps mainly to its β strand-rich DBD, which is consistent with the known client motif-binding preference of TRiC.

### Stat3 Binds to TRiC Subunit CCT3

**Figure 7:**
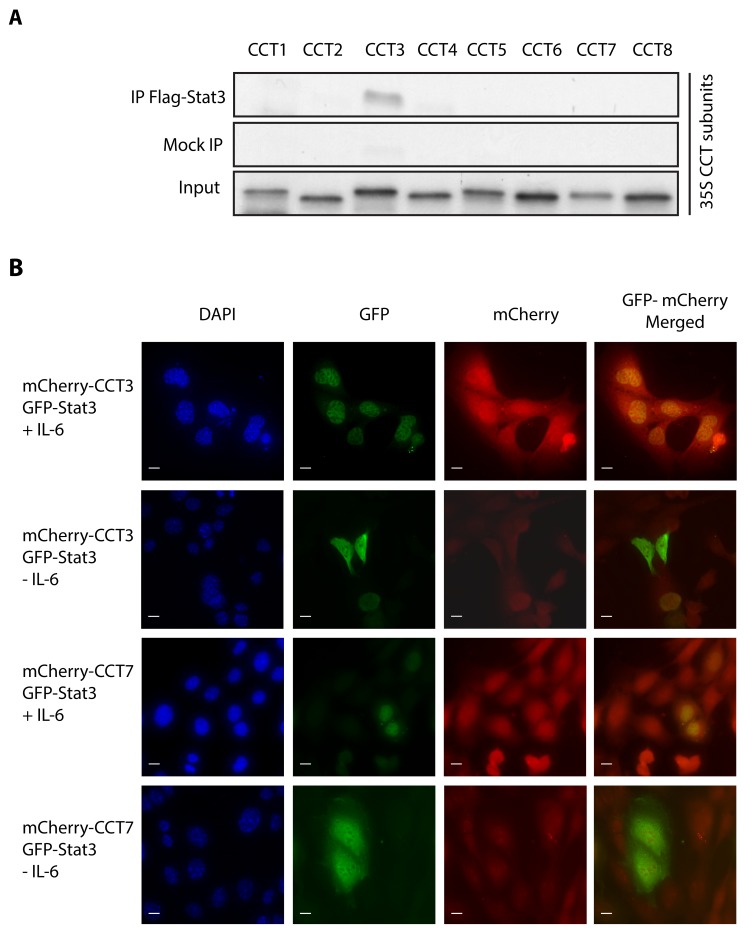
Stat3 binds and cross-links to TRiC subunit CCT3 in vitro; CCT3 co-localizes with activated Stat3 within the cell nucleus. In panel (A), RRLs expressing the indicated ^35^S-methionine-labeled TRiC subunit were mixed with RRLs expressing an unlabeled Flag-tagged Stat3 construct (Flag-Stat3) or no expression construct (Mock), incubated with DSP, immunoprecipitated with M2 anti-Flag antibody-bound agarose beads, incubated with β-mercaptoethanol, and analyzed by SDS-PAGE and autography. Autoradiographs are shown. In panel (B), transformed MEFs deficient in Stat3 and stably expressing GFP-Stat3α were transiently transfected with mCherry-CCT3 or mCherry-CCT7 and incubated without (*−*) or with (+) IL-6/sIL-6R (∼250 ng/ml), as indicated (±IL-6), before DAPI staining, fixation, and confocal fluorescent microscopic examination. Photomicrographs shown are representative images filtered to reveal the DAPI signal within the nucleus (column 1), the GFP signal within the cytoplasm and nucleus (column 2), the mCherry signal within the cytoplasm and nucleus (column 3), and the GFP plus mCherry merged signal (column 4). Bar, 10 µM.

There is compelling evidence that the subunits of TRiC differ in their substrate specificity, which suggests that Stat3 binding to TRiC likely is limited to a subset of CCT subunits [23]. To examine this hypothesis and to identify which CCT subunit(s) bind Stat3, we mixed RRLs expressing a single radiolabeled TRiC subunit with RRLs expressing either unlabeled Flag-tagged Stat3 or no specific protein; each RRL mixture was incubated with a reversible protein cross-linker (DSP) followed by immunoprecipitation with M2 anti-Flag antibody-bound agarose beads. The immunoprecipitated and cross-linked protein complexes were incubated with β-mercaptoethanol (to reverse the cross-linking) and analyzed by SDS-PAGE and autoradiography (Figure 7A). These studies revealed that only CCT3 consistently bound to and cross-linked with Stat3.

To confirm and extend these results *in vivo*, we transiently expressed CCT3 or CCT7 (negative control) each tagged with mCherry into MEF cells stably expressing GFP-tagged Stat3α (MEF/GFP-Stat3α) [25] and examined them by confocal fluorescent microscopy. We previously demonstrated that the intracellular distribution of GFP-Stat3α in MEF/GFP-Stat3α cells changed from predominantly cytoplasmic in unstimulated cells to predominantly nuclear upon IL-6 receptor stimulation [25]. Both CCT3 and CCT7 were distributed predominantly within the cytoplasm in co-transfected cells in the absence of IL-6 receptor simulation (Figure 7B). An increased fraction of CCT3, but not CCT7, co-translocated with GFP-Stat3α in the nucleus with IL-6 receptor stimulation, as assessed by image merging, which demonstrated a change in the color of the nucleus from green to yellow. We also performed ImagePro analysis to assess co-localization of GFP-Stat3α and CCT3 versus CCT7 in the absence or presence of IL-6 receptor stimulation. Using ImagePro to evaluate random microscopic fields, Pearson's correlation of co-localization within the nucleus for the CCT7 without IL-6 receptor stimulation (R_r_ = 0.512) did not change with IL-6 receptor stimulation (R_r_ = 0.493); in contrast, Pearson's correlation of co-localization within the nucleus for CCT3 increased markedly from R_r_ = 0.457 without IL-6 receptor stimulation to R_r_ = 0.800 with IL-6 receptor stimulation. In addition, ImagePro analysis of multiple fields of cells co-expressing GFP-Stat3α and either CCT3 or CCT7 and stimulated with IL-6/sIL-6Rα revealed a significantly higher Pearson's coefficient of co-localization within the nucleus for cells expressing CCT3 (0.90±0.06) versus CCT7 (0.62±0.21; *p* = 0.04).

### Addition of the pVHL TBD to Stat3 Strengthens Its Binding to TRiC and Improves Its Function

**Figure 8:**
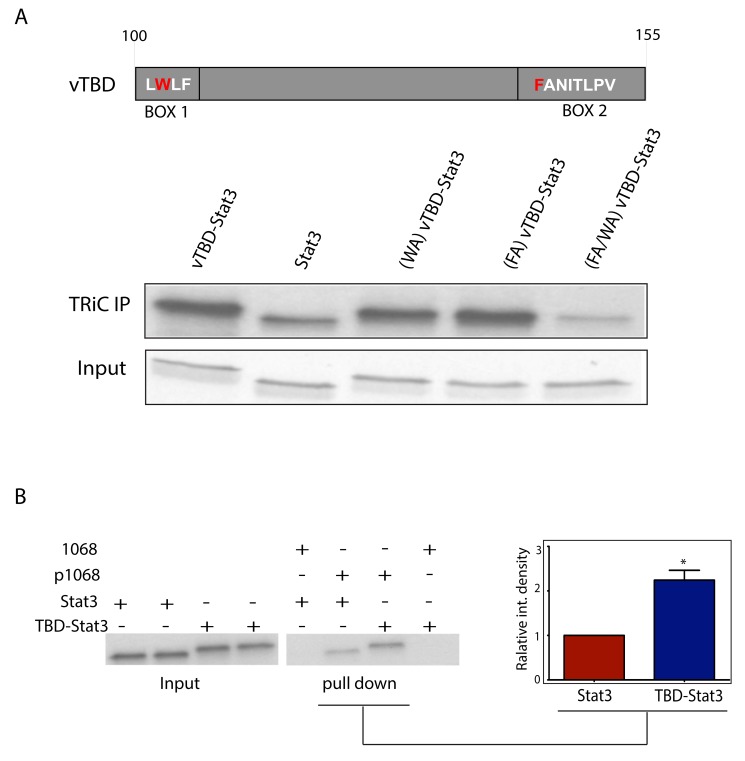
vTBD-Stat3 demonstrates increased binding to TRiC; increased binding is mediated by residues within the vTBD Box 1 and 2 and is accompanied by improved SH2 domain function. The top portion of panel (A) delineates the vTBD including the location of Box 1 and Box 2 and critical residues therein including those shown in red, which were mutated to alanine. Following translation of the indicated proteins in the presence of ^35^S-methionine, TRiC was immunoprecipitated from RRLs with a combination of antibodies to CCT2 and CCT5 (TRiC-IP). Immunoprecipitates were separated by SDS-PAGE and autoradiographed (middle portion). Autoradiography of an equivalent volume of each immunoprecipitation reaction pair (Input) is shown in the lower portion. In panel (B), equivalent amounts of ^35^S-methionine labeled Flag-Stat3 or Flag-vTBD-Stat3 (Input) were incubated with biotinylated tyrosine phosphorylated dodecapeptide 1068 (p1068) or unphosphorylated dodecapeptide (1068) bound to streptavidin agarose beads. Bead-bound proteins (pull down) were separated by SDS-PAGE and autoradiographed. Autoradiographs of Input (left panel) and Pull Down (middle panel) proteins of a representative of three experiments are shown. Densitometry of the underlined bands from all experiments were normalized to the Stat3 in experiment 1 and the mean ± SEM plotted (right panel). The asterisk (*) indicates increased binding to p1068 by vTBD-Stat3 versus Stat3 (*p* = 0.0028).

The TBD of pVHL previously mapped to a 55 amino-acid residue, β-strand-rich [23] region within pVHL; addition of the TBD of pVHL (vTBD) to the N-terminus of dihydrofolate reductase (DHFR), a protein that does not naturally bind to TRiC, conferred TRiC binding [23]. We hypothesized that adding the vTBD to Stat3 would increase binding of the resultant hybrid protein, vTBD-Stat3, provided the vTBD bound to TRiC via CCT subunits that are distinct from those that bind the TBD of Stat3 (sTBD); pVHL binding to TRiC previously mapped to CCT1 and CCT7 [26]. To test this hypothesis, we placed the vTBD at the N-terminal end of Stat3 and examined its ability to bind to TRiC compared to wild-type Stat3. The amount of vTBD-Stat3 that co-precipitated with TRiC from RRLs was increased over 2-fold compared to wild-type Stat3 (Figures 1A and 8A). Binding of the vTBD to TRiC previously mapped to specific residues within two β strands located at the N-terminal end (Box 1) and C-terminal end (Box 2) of the vTBD [23],[26]. A combination of point mutations of critical residues within Box 1 and 2 of the vTBD portion of vTBD-Stat3 reduced its ability to co-immunoprecipitate with TRiC to levels similar to those of wild-type Stat3 (Figure 8A). Thus, addition of the vTBD to Stat3 increased its binding to TRiC; the increased binding, not unexpectedly, was mediated through Box 1 and Box 2 within vTBD.

To assess if increased interaction of Stat3 with TRiC affects the function of Stat3, we compared the activity of *in vitro* translated vTBD-Stat3 to wild-type Stat3. We previously demonstrated that Stat3 bound with high affinity to a phosphotyrosyl-dodecapeptide (p1068) through its Src-homology (SH) 2 domain [27]. This dodecapeptide is based on residues 1063 to 1074 within the epidermal growth factor receptor and contains a Y residue at position 1068, which when phosphorylated was shown to recruit Stat3 [27]. Phosphopeptide pull-down assays demonstrated a 2.3-fold increase in the amount of vTBD-Stat3 that binds to phosphopeptide p1068 compared to wild-type Stat3 (*p*<0.0028; Figure 8B). These results strongly suggest that addition of the vTBD to Stat3 not only increases its interaction with TRiC but also increases the fraction of SH2 domains within vTBD-Stat3 that are fully folded and functional compared to wild-type Stat3.

## Discussion

Our understanding of how transcription factors achieve their folded and functional conformations within cells is incomplete. Here we demonstrate Stat3, a transcription factor activated in up to 50% of cancers, binds TRiC/CCT chaperonin and that TRiC is required for Stat3 biosynthesis and activity *in vitro* and within cells. TRiC binding to Stat3 was mediated, at least in part, by TRiC subunit CCT3. Stat3 binding to TRiC mapped primarily to its β-strand rich, DBD. Addition of a second TBD vTBD further increased its affinity for TRiC, as well as its function. Thus, Stat3 levels and function are regulated by TRiC and can be modulated through manipulation of substrate affinity and TRiC levels within the cell.

Approaches used successfully to identify TRiC clients have included both directed and global strategies. The earliest recognized TRiC clients—β-actin [28], the α and β subunits of tubulin [29]–[31], and firefly luciferase [31] —were identified in TRiC complexes using nondenatured gel electrophoresis and co-immunoprecipitation of denatured recombinant proteins in solution or from RRLs. Additional TRiC clients were subsequently identified by (i) co-elution or co-immunoprecipitation with TRiC in RRLs (actin-related protein and γ-tubulin [32], G-transducin [33], myosin heavy chain [34], and histone deacetylase 3 [35]), (ii) TRiC binding in native gels (cofilin, actin-depolymerization factor-1, cofactor A, Cyclin B, cap-binding protein, CCT1, H-ras, and c-Myc [36],[37]), (iii) yeast two-hybrid screens or co-immunoprecipitation with TRiC from yeast cell lysates (cyclin E, [38], Cdc20 and Cdh1 [39], polo-like kinase [40], and sphingosine kinase 1 [41]), (iv) co-immunoprecipitation with TRiC following overexpression in human cell lines (pVHL, [42]), and (v) mass spectrometry analysis to detect TRiC/CCT peptides within selected immunoprecipitates (p53, [43]).

Taking a more global approach [13],[24],[44], we recently used pulse-chase analysis involving TRiC immunoprecipitation, 2-D gel electrophoresis, and mass spectrometry to identify highly abundant TRiC clients including the WD repeat-containing translation initiation factor-3 and GAPDH [13]. In addition, we screened 2,600 clones from a murine C2C12 cell cDNA library using small-pool expression cloning in RRLs to identify low-abundant TRiC clients [13]. This approach identified 167 clients including proteins involved in cell cycle, cytoskeleton, protein degradation, DNA metabolism, meiosis, mitosis, carbohydrate metabolism, RNA processing, signal transduction, protein trafficking, transcription, and translation. Stat3 was not among the 167 TRiC clients identified in this screen most likely because of its lower abundance in the cell compared to other TRiC clients such as actin and tubulin and also due to the limited number of cDNAs screened.

Our report represents the most comprehensive demonstration that an oncoprotein, Stat3, is a TRiC client. As noted above, earlier investigators [36] demonstrated binding of radiolabeled, denatured H-ras and c-Myc binding to TRiC in native gel assays. However, the affinity of binding was low, the results were not confirmed by other methods, and the contribution of TRiC binding to H-ras or c-Myc biogenesis and function was not examined.

There have been few studies that simultaneously compare the effects of targeting TRiC on the levels and function of multiple TRiC clients. In this regard, it is noteworthy that despite 90% reduction of TRiC within cells, levels of the TRiC clients, β-actin, GAPDH, and β-tubulin were not reduced (Figures 4 and S1, and unpublished data), in contrast to levels of pStat3 and total Stat3 (Figure 4). These results suggest a stricter requirement for TRiC to assist in Stat3 biogenesis and folding versus β-actin, GAPDH, or β-tubulin.

Earlier reports have supported a contribution of chaperones to the biology of Stat3. Stat3 previously was identified within the cytosol of unstimulated cells (HepG3), predominantly within high-molecular weight complexes in the size range of 200–400 kDa (Statosome I) and 1–2 MDa (Statosome II) [45]. Antibody-subtracted differential protein display using anti-Stat3 antibody identified the chaperone GRP58/ER-60/ERp57 as a major component, along with Stat3, of Statosome I [45]. The composition of Statosome II was not determined; however, it is of interest, in light of our current findings, that its size is consistent with that of TRiC. Stat3 was demonstrated to directly interact with Hsp90 [46],[47], and its activation was linked to Hsp90 following IL-6 stimulation [48]. In addition, inhibition of Hsp90 using pharmacological inhibitors (17-DMAG) or siRNA decreased levels of pStat3 in human primary hepatocytes and levels of total Stat3 in multiple myeloma cells.

We have shown by immunoprecipitation studies in RRLs that the interaction between TRiC and Stat3 occurs predominantly through the CCT3 of TRiC binding to the DBD of Stat3. Other domains of Stat3, including the N-terminal domain, linker domain, and SH2 domain, also were pulled down with TRiC, although at lower levels than the DBD indicative of lower affinity interactions. Of these domains, only the SH domain contains β-strands.

We demonstrate in this report that chaperonin–client interactions can be modulated not only to reduce client function but also to increase it. Knockdown of TRiC using shRNA reduced total Stat3 protein levels, reduced constitutively activated Stat3, and reduced the sensitivity of Stat3 to IL-6–mediated activation. These findings provide proof-of-principle that targeting TRiC, or more specifically targeting the interaction between TRiC and Stat3, may provide a novel approach to reducing levels of activated Stat3 for therapeutic benefit in cancer. Alternatively, addition of a new TRiC binding site to Stat3 through a covalent modification significantly improved its affinity for TRiC and its function. This result serves as proof-of-concept that future development of noncovalent modulators that increase TRiC–Stat3 interactions may be a novel approach to increasing Stat3 activity in the setting of acute injuries such as myocardial infarct [49] or traumatic injury where enhanced Stat3 has been demonstrated to prevent apoptosis of parenchymal cells within critical organs including cardiomyocytes, alveolar epithelial cells, and hepatocytes [50]–[52].

## Materials and Methods

### Cell Lines

All cell lines were maintained at 37°C in 5% CO_2_ in DMEM with 10% fetal bovine serum, 1% penicillin/streptomycin, and Glutamax. MEF/Stat3^Δ/Δ^ cells were provided as a generous gift from Dr. Valeria Poli [17]. MEF/Stat3^Δ/Δ^ cells stably transfected with GFP-Stat3α were generated as described [53]. HepG2 and HS578T cells in which TRiC was stably knocked down were generated by lentiviral transduction of shRNA targeting TCP1β (CCT2) within lentiviral transduction particles used according to the manufacturer's protocols (Sigma-Aldrich) and selection with puromycin.

### Plasmids

The coding sequence for human Flag-tagged pVHL and Stat3 were subcloned into a modified pSG5 vector as XhoI/HindIII and HindIII/NotI fragments, respectively. Stat3 was also engineered to contain the 55 amino-acid TBD of pVHL (vTBD) at the N-terminal end to generate vTBD-Stat3. The sequence encoding the 55 amino acids was generated by PCR using VHL cDNA as template and subcloned into the mammalian expression vector pSG5. For mapping the TBD within Stat3, sequences encoding each of the six domains of Stat3 were generated by PCR and cloned into pSG5 vector. All inserts were sequenced to ensure fidelity.

### *In Vitro* Transcription and Translation

*In vitro* transcription and translation reactions (50 µl) were carried out using an RRL system (TNTT7 Quick Coupled Transcription/Translation System; Promega, Madison, WI) with 1 µg of pSG5 vector containing the insert of interest according to the manufacturer's instructions for 30 min at 30°C, and terminated by the addition of 2 mM puromycin, 5 mM EDTA, and 1 mM azide. Protein translation was monitored by SDS-PAGE followed by autoradiography. Where indicated, RRLs were depleted of ATP by incubation for 10 min at RT with apyrase (50 U/ml).

### TRiC Immunoprecipitation, Immunodepletion, and Immunoblotting

TRiC immunoprecipitations were performed as described [42]. Briefly, 10 µl of each RRL reaction was diluted to 50 µl with buffer (25 mM Tris, pH 7.5, 10% glycerol, 5 mM ETDA, 100 mM NaCl, 1 mM azide) and treated with antibodies to TRiC (mixture of rabbit anti-CCT2 and anti-CCT5) or rabbit anti-human IgG control antibody (Abcam, Cambridge, MA) for 45 min on ice. The mixture was incubated with 30 µl of Protein A-Sepharose and gently agitated on ice for 40 min. The immune complexes were pelleted and then washed 4 times with buffer containing 0.5% Triton X-100. The protein samples were separated by SDS-PAGE and visualized by autoradiography. For immunodepletion of TRiC, 6 µg of rabbit anti-CCT1 antibody or rabbit anti-human IgG control antibody was added to RRLs and incubated at 4°C for 4 h. The immune complexes were bound by Protein A-agarose and removed by centrifugation. For immunoprecipitation analysis of Stat3/TRiC interactions in mammalian cells, murine embryonic fibroblast cells in which Stat3 was deleted using Cre/Lox technology (MEF/Stat3^Δ/Δ^ cells) [17] were plated in 100 mm plates and transfected with PSG5 Flag-Stat3 construct using GeneJuice transfection reagent per the manufacturer's instructions (EMD Millipore); 48 h after transfection, the cells were washed and harvested in ice-cold PBS or ATP depletion buffer (PBS containing 2 mM deoxyglucose, 1 mM sodium azide, and 5 mM EDTA). Lysates were prepared by dounce homogenization (70 strokes; pestle B) in 1 ml of lysis buffer A (20 mM HEPES, pH 7.5, 100 mM NaCl, 5 mM EDTA, 5% glycerol) or Buffer B without 5 mM EDTA, containing instead 2 mM ATP and 5 mM MgCl_2_. The lysates were either used immediately or snap frozen in liquid nitrogen and stored at −80°C. Lysate (100 µl) was incubated on ice for 1–2 h with 3 µl each of rabbit antibodies to CCT2 and CCT5 or rabbit IgG anti-human control antibody. Magnetic protein A beads (50 µl equivalent; Invitrogen) were resuspended in the antibody/lysate mixtures and incubated for 10 min on ice. Beads were washed two times with 500 µl of buffer A and two times with buffer A containing 1% Triton ×100. Bound proteins were separated by SDS-PAGE and immunoblotted.

### Stat3-TRiC Subunit Cross-Linking Studies

RRL reactions containing ^35^S-methionine-labeled TRiC subunits were mixed with RRLs containing either an unlabeled Flag-tagged Stat3 construct or no expression construct; each RRL mixture then was incubated with the reversible protein cross-linker DSP (dithiobis[succinimydyl-propionate]) at a final concentration of 2 mM. Samples were quenched by 10-fold dilution in 20 mM Tris pH 7.5 containing 10% glycerol prior to immunoprecipitation with M2 anti-Flag antibody-bound agarose beads. The immunoprecipitated and cross-linked protein complexes then were incubated with β-mercaptoethanol (5 mM) to reverse the cross-linking and analyzed by SDS-PAGE and autoradiography.

### Phosphopeptide Binding Assay

Biotin-dodecapeptide 1068 or biotin-phosphododecapeptide p1068 (10 µl at 10 mg/ml) were synthesized commercially based on the sequence within the cytoplasmic portion of the EGFR surrounding Y1068 previously shown to bind Stat3 through its SH2 domain [27]. Each peptide was added to 100 µl of 50% (v/v) Neutravidin beads (Thermo Fisher Scientific, Rockford, IL) in PBS and incubated overnight. The beads were washed 3 times with PBS and resuspended in 100 µl buffer 25 mM HEPES pH 7.5 containing 20% glycerol, Phostop (Roche), and protease inhibitor cocktail (Sigma-Aldrich, St. Louis, MO). RRL volumes were adjusted to match input protein concentration based on densitometry results. The lysates were diluted 50-fold in 100 µl buffer and treated with Neutravidin beads immobilized with either p1068 or control 1068 dodecapeptide. The samples were incubated for 1 h at 4°C. The beads were then washed 4 times with 500 µl of HEPES buffer containing 0.5% NP40 and analyzed by SDS-PAGE and autoradiography.

### TRiC Knockdown in Cells

To reduce TRiC levels within cells, MISSION shRNA lentiviral transduction particles targeting CCT2 sequence (SHCLNV-NM-006431) and control shRNA lentiviral particles were obtained from Sigma-Aldrich. HepG2 and HS-578T cells were transduced with CCT2 shRNA or control shRNA lentiviral particles at an MOI of 5 and grown for 48 h. Stable TRiC knockdown cells were established by replating the cells in selection media containing puromycin at 3 µg/ml.

### IL-6 Stimulation of Cells

CCT2 knockdown or control HepG2 cells were cultured in six-well plates overnight in DMEM supplemented with 10% fetal bovine serum (FBS, GIBCO-BRL, Invitrogen, Carlsbad, CA) containing 2 mM Glutamax (GIBCO-BRL), 10 mg/ml penicillin, and 10 mg/ml streptomycin (GIBCO-BRL) at 37°C in 5% CO_2_. The cells were then treated with IL-6 at 0, 0.1, 0.3, and 1 ng/ml for 30 min at 37°C, then lysed in M-PER lysis buffer (Thermo Fisher Scientific) supplemented with phosphatase and protease inhibitor cocktails.

### Measurements of Y705 Phosphorylated (p)Stat3, Total Stat3, and GAPDH by Immunoblotting and Luminex Bead-Based Assays

For immunoblotting, total protein concentration was measured within M-PER lysates using the Coomassie assay kit (Bio-Rad, Hercules, CA) with BSA as a standard. Total cell lysates containing 20 µg of total protein were separated by 7.5% SDS-PAGE and transferred onto PVDF membranes. The membranes were blocked with 5% BSA in 0.1% TBS-T (0.1% Tween-20 in TBS) for 1 h and incubated overnight at 4°C in blocking buffer with one of the primary antibodies against total Stat3, pStat3 (BD-Biosciences), β-actin, GAPDH, CCT1, CCT2, and CCT5 (Abcam); washed; incubated with appropriate secondary antibodies conjugated to horseradish peroxidase; washed; and developed with ECL substrate (Thermo Scientific). For Luminex bead assays, cells were lysed and assayed for pStat3, total Stat3, and GAPDH using Millipore (Milliplex) kits as described by the manufacturer. Levels of each protein analyte were determined and analyzed using the Bio-Plex suspension array system (Bio-Rad).

### Fluorescence Microscopy

Transformed MEF cells stably expressing GFP-Stat3α(MEF/GFP-Stat3α) were plated on polylysine-coated glass coverslips. After 12 h the cells were transfected with mCherry-CCT3 or mCherry-CCT7 fusion constructs using GeneJuice reagent (EMD4 Biosciences) according to the manufacturer's instructions. Forty-eight hours later, cells were treated with or without IL-6/sIL-6R (∼250 ng/ml) for 30 min. The coverslips were rinsed with ice-cold PBS twice and fixed for 30 min with 4% paraformaldehyde in PEM buffer (80 mM potassium PIPE, pH 6.8, 5 mM EGTA, and 2 mM MgCl_2_) at 4°C, rinsed 3× with PEM buffer, quenched in 1 mg/ml NaBH4 (Sigma) in PEM buffer for 5 min, then permeabilized with 0.05% Triton X-100 in PBS for 5 min, counterstained with 4′,6-diamidino-2-phenylindole (DAPI, Invitrogen), mounted onto slides, and imaged using confocal fluorescence microscopy.

### Protein Purifications

TRiC was purified from bovine testes essentially according to previously established procedures [23],[54]. To achieve extra purity and remove co-purifying bound substrates, prepared TRiC fractions were incubated for 15 min at 37°C in the presence of 1 mM ATP and then re-applied to MonoQ HR 16/10 (GE Healthcare, USA) and Superose 6 10/300 GL column (GE Healthcare, USA) in sequence. Ultra-purified TRiC retained its full capacity to fold substrate as assessed in a luciferase refolding assay performed as described [55]. The sequence encoding the human full-length Stat3 was expressed in *Escherichia coli* as a fusion to a C-terminal His_10_-tag in pET28a (Novagen) expression vector. The transformed cells were induced with 1 mM isopropyl-β-d-thiogalactopyranoside (IPTG) at an optical density of 0.7 at 600 nm. After growing for 5 h at 37°C, cells were harvested; resuspended in buffer containing 20 mM Tris-HCl, pH 8.0, 150 mM NaCl, 1 mM DTT, 1% Triton X-100, and 100 µg/ml lysozyme; and lysed by sonication. The insoluble fraction containing Stat3 as inclusion bodies was collected by centrifugation and washed extensively with a buffer containing 2 M urea. The washed pellet was solubilized in 0.1 M NaH_2_PO_4_, 10 mM Tris-HCl, pH 8.0, 300 mM NaCl, 5 mM imidazole, 10% glycerol, and 6 M guanidinium hydrochloride (Gn-HCl). After centrifugation, the supernatant was applied onto a Ni-NTA column (Qiagen) equilibrated in the same buffer. The column was washed successively with buffer A (0.1 M NaH_2_PO_4_, 10 mM Tris-HCl, pH 6.3, 300 mM NaCl, 6 M Gn-HCl) and buffer B (buffer A at pH 5.9). Proteins were eluted from the column with buffer C (buffer A at pH 4.5), concentrated, and buffer exchanged into buffer C containing 1 mM DTT. Proteins were stored at −80°C until use.

## Supporting Information

Figure S1 Targeting of CCT2 within cells using shRNA reduces levels of other CCT subunits. HS-578T cells stably expressing CCT2 or control shRNA were immunoblotted for the indicated proteins using specific antibodies to each and the results of a representative experiment shown.
(TIF)

## Abbreviations

BSA: bovine serum albumin
CCT: chaperonin containing TCP-1
DBD: DNA-binding domain
GFP: green fluorescent protein
MEFs: mouse embryonic fibroblasts
pVHL: von Hippel-Lindau protein
RRLs: rabbit reticulocyte lysates
Stat3: signal transducer and activator of transcription 3
TBD: TRiC binding domain
TRiC: tailless complex protein-1 (TCP-1) ring complex
