# Supplementary figures and images for: Modulation of STAT3 Folding and Function by TRiC/CCT Chaperonin
Source: PLoS Biol. 2014 Apr 22;12(4):e1001844. doi: 10.1371/journal.pbio.1001844 (PMC3995649; doi:10.1371/journal.pbio.1001844)

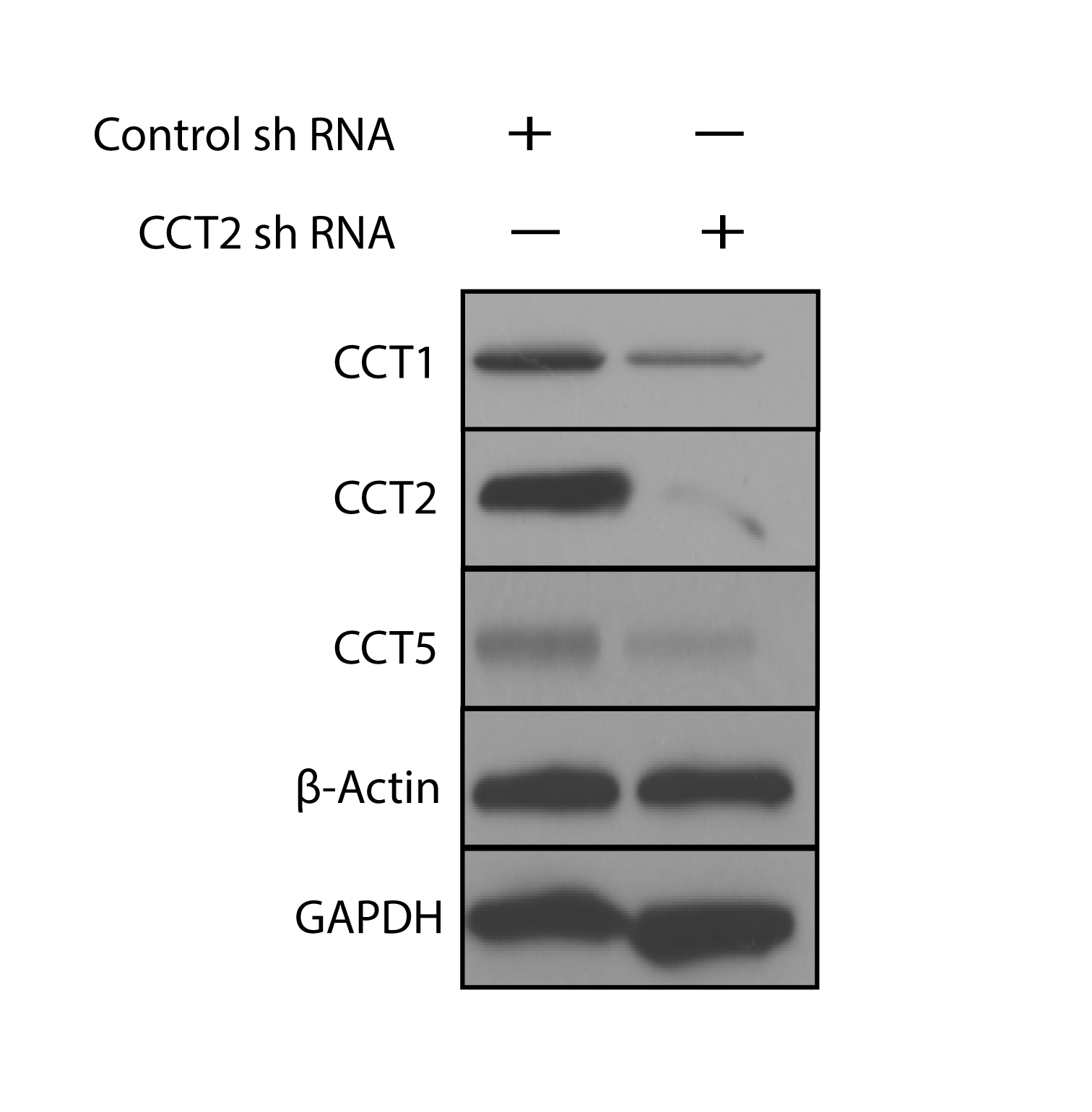

Supplement: Figure S1 — Targeting of CCT2 within cells using shRNA reduces levels of other CCT subunits. HS-578T cells stably expressing CCT2 or control shRNA were immunoblotted for the indicated proteins using specific antibodies to each and the results of a representative experiment shown. (TIF) [file pbio.1001844.s001.tif]
